# Nurse‐led mental and physical healthcare for the homeless community: A qualitative evaluation

**DOI:** 10.1111/hsc.13778

**Published:** 2022-03-09

**Authors:** Lauren Bell, Maxine Whelan, Emily Fernandez, Deborah Lycett

**Affiliations:** ^1^ 2706 Centre for Intelligent Healthcare Coventry University Coventry UK; ^2^ Warwickshire County Council Warwick UK

**Keywords:** access to healthcare, community health care, health inequalities, homelessness, rough sleeping

## Abstract

Increased morbidity and mortality rates are prominent issues among homeless individuals. To help reduce these health inequalities, dedicated senior mental and physical health nurses have been deployed to work within and alongside local statutory and voluntary organisations. This qualitative evaluation examined the impact of nurse‐led homeless healthcare in Warwickshire, United Kingdom. During January and February 2021, online semi‐structured interviews were conducted with 17 professionals including the mental and physical homeless health nurses (*n* = 4), statutory health and local authority professionals (*n* = 4), and voluntary and community sector professionals (*n* = 9). Interviews were qualitatively analysed using inductive, reflexive thematic analysis. Data analysis identified three overarching themes related to the meaning, impact and future development of nurse‐led homeless healthcare: (1) Nurse‐led homeless healthcare and health inequalities, (2) The multi‐agency approach of nurse‐led homeless healthcare, and (3) Future development of nurse‐led homeless healthcare. The findings confirm the benefits of homeless healthcare in reducing health inequalities and promoting a more accessible, flexible and person‐centred approach to holistic care. Yet, prevailing organisational and system‐level barriers were also identified as currently limiting the capacity, provision and practicalities of delivering nurse‐led homeless healthcare. Recommendations were identified with international relevance and included: (i) continued implementation of person‐centred healthcare for homeless individuals, (ii) strengthening of organisational collaboration and communication pathways to improve coordinated care, (iii) development of the managerial and structural aspects of provision, (iv) addressing limitations associated with scope and capacity to ensure that delivered healthcare is adequately intensive, (v) increased availability of clinical or therapeutic spaces, and (vi) implementation of long‐term plans supported by evaluation and commissioning.

**What is known about this topic**

- Mental and physical health inequalities among homeless individuals have been internationally evidenced.
- National policies and local homelessness prevention strategies aim to reduce health inequalities and improve health through integrating health, care, and support services.

**What this paper adds**

- Nurse‐led homeless healthcare was indicated to increase access to mental and physical healthcare and may reduce health inequalities.
- Effective support for homeless individuals is strengthened by multi‐agency partnerships across statutory and voluntary and community organisations.
- Strong referral pathways and structures at organisational and system levels were important in building client engagement with both homeless healthcare and wider services and to ensure a professionally coordinated approach.

## 1 INTRODUCTION

Health inequalities are prevalent among homeless individuals. Increased morbidity and mortality are associated with accident and injury, suicide and long‐term conditions including infectious and cardiovascular diseases (Aldridge et al., 2018; Fazel et al., 2014; Field et al., 2019; Queen et al., 2017). Poverty, trauma, substance misuse and mental illness also contribute (Aldridge et al., 2018; Fazel et al., 2014; Field et al., 2019; Queen et al., 2017). Barriers to accessing healthcare include stigma, social exclusion and unsuitable communication pathways (Elwell‐Sutton et al., 2017; Gunner et al., 2019; Magwood et al., 2020), which can increase emergency care utilisation (Mitchell et al., 2017; Rae & Rees, 2015). Individuals may also experience co‐occurring mental illness and substance misuse, referred to as ‘dual diagnosis’ (Cream et al., 2020), however individuals may be ineligible for specialist services because of these comorbidities (Carrà et al., 2015).

Additional provisions have been designed to better care for homeless individuals (Bryar, 2020; Gunner et al., 2019). Projects have utilised community spaces such as libraries to deliver healthcare (Mariano & Harmon, 2019) and observed increased engagement with primary care when in contact with a community health nurse (Su et al., 2015). Positive implications for identifying and treating health issues and reducing emergency care have also been identified (Bryar, 2020; Cream et al., 2020). Homeless healthcare, however, can be restricted by temporary commissioning, and only half of homeless projects (e.g., hostels) in the United Kingdom (UK) are linked to specialist health services (Crane et al., 2018). Less than 50% of UK mental health services report dedicated resources for homeless patients, and staff training in homelessness is limited (Lucas et al., 2018). The development, implementation and evaluation of homeless healthcare is therefore ongoing.

Authorities in the UK have implemented guidance and strategies to reduce homelessness and improve health (Public Health England, 2019). Central to this is multi‐agency partnerships between statutory services (services provided by local and national government, including health and social care) and voluntary and community organisations, which are purported to improve holistic and coordinated healthcare and subsequently reduce inequalities (Cream et al., 2020; Luchenski et al., 2018). The voluntary sector have traditionally played a significant role in supporting homeless individuals, using more flexible methods than those observed in statutory healthcare (Flanagan & Hancock, 2010), offering opportunities for developing homeless healthcare.

### 1.1 Context for this evaluation

**FIGURE 1:**
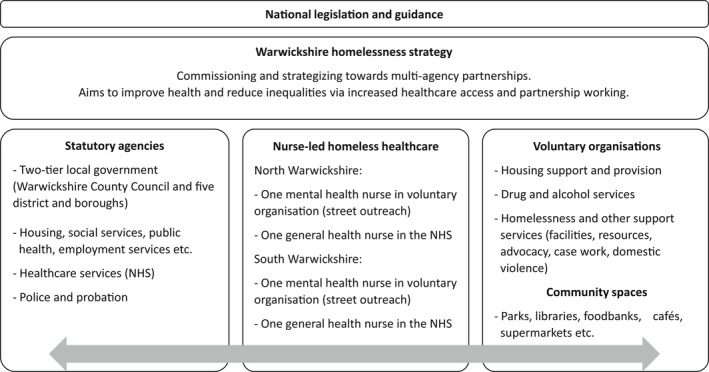
Structure of the nurse‐led homeless healthcare and types of partner organisations working in national and local strategies. Note: National Health Service (NHS)

This study evaluates nurse‐led homeless healthcare delivered in Warwickshire, UK, a rural county that reports increased rates of homelessness and recent counts of 47 individuals rough sleeping (e.g., on the street or in tents) (Public Health England, 2021; Warwickshire County Council, 2021). Rural homelessness poses challenges for reaching more isolated individuals, delivering specialist services over greater distances and implementing effective commissioning (Snelling, 2017). In 2019, mental health nurses (experienced in psychological therapies and motivational interviewing) were seconded into a voluntary organisation delivering community‐based housing support (Figure 1). This structure aimed to develop street‐based and outreach approaches to homeless healthcare for individuals rough sleeping (in year one of the pilot) and those vulnerably housed (from year two). General nurses have delivered physical health provision (e.g., wound care, blood‐borne virus treatments) since 2020 and remained employed by the National Health Service (NHS). The full‐time homeless health nurses are senior and have specialist expertise in their respective fields, where prior experience with homeless patients was only that gained from working in public healthcare. Akin to ‘street medicine’, where healthcare is delivered in locations more accessible to prospective patients (Stefanowicz et al., 2021), the nurse‐led homeless healthcare pilot aimed to deliver person‐centred care to improve health, strengthen system‐wide partnerships and disseminate professional knowledge across the multi‐disciplinary team. In this article, ‘clients’ is used to denote individuals who may access homeless healthcare.

### 1.2 Aims

The specific aims of this evaluation were to understand (i) how the mental and physical health of clients was supported by nurse‐led homeless healthcare, including via access to other services and (ii) professionals’ experiences of collaborative working and whole‐system outcomes, including the impact of nurse‐led homeless healthcare on organisational partners.

## 2 METHODS

### 2.1 Design

In this qualitative study, semi‐structured interviews were conducted with professionals working with homeless individuals in Warwickshire. The design was discussed with key professionals to ensure appropriateness. Ethical approval was received from Coventry University (p110165). This evaluation is written according to COREQ requirements (Tong et al., 2007).

### 2.2 Sample and recruitment

In North and South Warwickshire, nurse‐led homeless healthcare was delivered by one mental health nurse and one general nurse (four nurses in total across Warwickshire). Via joint casework and multi‐agency meetings, the homeless health nurses worked alongside statutory services (e.g., housing, health, police and probation) and the voluntary and community sector (e.g., housing support and drug and alcohol services), as depicted in Figure 1. Eligible participants contributing to this evaluation therefore included professionals working for or alongside nurse‐led homeless healthcare in Warwickshire.

During COVID‐19, interviews were conducted remotely between January and February 2021. Relevant gatekeepers disseminated Participant Information Sheets to key organisational contacts, and snowball sampling identified further participants. Interested individuals contacted the research team, and all participants provided informed consent using survey software (Qualtrics). No participant withdrew, however with this recruitment approach response rates are unknown. We also intended to interview clients. Regrettably, clients were not interviewed due to challenges accessing confidential spaces during COVID‐19.

### 2.3 Reflexivity

Interviews were conducted by a Research Assistant with a public health research interest and experience conducting research interviews. Participants were informed that the research institution was not involved in the design or delivery of the homeless healthcare pilot, and that participants would not be explicitly identified alongside quotes. However, participants were from a limited potential pool, and this was recognised as a factor that could influence responses. Analysis therefore applied a critical realist perspective, whereby participants were assumed to present meaningful and individual experiences and perspectives, though participant responses may be filtered or modified in the interview context (Fletcher, 2017).

### 2.4 Data collection

Only the interviewer and participant were present during interviews. Participants chose where to complete the interview, such as their home or workplace. According to a semi‐structured schedule, participants reported their perceptions of nurse‐led homeless healthcare and its impact on clients, organisational collaboration, knowledge‐sharing, referral pathways and recommendations for homeless healthcare. Follow‐up questions sought clarification and in‐depth understanding. All interviews were audio‐recorded with consent. Field notes were not produced and interviews were not repeated. Participants were not invited to comment on transcripts.

### 2.5 Data analysis

Audio recordings were transcribed verbatim and identifiable information removed. Participants were informed that quotes would be presented with pseudonyms and their own pseudonym could be chosen. Quotes are not presented with participants’ professional roles to support anonymity. Verbatim quotes have been modified to aid readability (e.g., removing word repetitions and hesitations).

A reflexive thematic analysis was conducted according to a six‐phase approach (Braun & Clarke, 2006, 2020). After data familiarisation, LB assigned codes to all transcripts inductively (i.e., data driven) and without automated software. A second researcher (MB) examined three transcripts to offer alternative interpretations, codes and to support reflexivity. LB organised codes into candidate themes, which were then collaboratively refined among the researchers. Themes and definitions were revised throughout the writing process, and quotes were chosen to illustrate patterns and represent the range of participants. Independent coding and saturation were not concepts aligned with the chosen reflexive approach (Braun & Clarke, 2021).

## 3 RESULTS

### 3.1 Participants

A total of 17 professionals (see Table 1) participated in interviews that lasted *M* = 38 min (range 20–67 min).

**TABLE 1:** Professional roles of participants

Professional role	Number (*n*)
Homeless health nurses	4
Local authority housing professionals	2
Mental health professionals	2
Substance misuse professionals	3
Housing support professionals	2
Support services for homeless or vulnerably housed individuals	4

### 3.2 Qualitative findings

Thematic analysis identified three master themes: (1) Nurse‐led homeless healthcare and health inequalities, (2) The multi‐agency approach of nurse‐led homeless healthcare, and (3) Future development of nurse‐led homeless healthcare.

#### 3.2.1 Theme 1: Nurse‐led homeless healthcare and health inequalities

This theme reports the perceived barriers to accessing mainstream healthcare while homeless, (subtheme 1A), how nurse‐led homeless healthcare impacts access through a person‐centred approach (subtheme 1B), and perceived holistic, biopsychosocial changes in clients (subtheme 1C).

##### Subtheme 1A: Barriers to mainstream healthcare

Many participants justified the need for dedicated homeless healthcare because of barriers that people who are homeless face in mainstream healthcare. Usual healthcare registration and patient‐provider communication pathways were described as inflexible and inappropriate for homeless individuals: ‘‘*if they don't have internet access, then they're not able to book the appointments online. Or they don't have the credit on their phone’’* (Katie). Homeless individuals were also perceived to experience stigma in mainstream healthcare: ‘‘*they feel judged, they feel like second class citizens, and that's being reinforced isn't it, by the message that if you don't have the things that a normal homeowner has, then you can't register here*’’ (Sharon).

Inappropriate eligibility criteria for mainstream services also prevented holistic healthcare for issues such as dual diagnosis: ‘‘*one service might say well that [substance misuse] needs to be addressed before we can look at the mental health side’*’ (Ian). Waiting times for appointments or treatments were further unsuitable for individuals with acute needs: ‘‘*it's no good saying make an appointment in three days if somebody's having a psychotic episod*e’’ (Michelle). Taken together, participants widely noted that people who are homeless face challenges with mainstream healthcare, and that appropriate homeless healthcare was required to address known health inequalities.

##### Subtheme 1B: Improving access to healthcare via a person‐centred approach

Participants appraised nurse‐led homeless healthcare as reducing many barriers present in mainstream healthcare. Delivering compassionate care and building trusting patient–professional relationships was an important feature: ‘‘*what the [homeless health] nurses have is the ability to say, ‘I'm here for you and for the challenges that you face’’’* (Donna). The person‐centred approach meant that the homeless health nurses were more prepared and responsive to clients’ varying needs: *“people aren't always the best at appointments and sitting in a room…when the nurses have been most effective is when they've been able to work flexibly at street level.”* (Adrian). Similarly, the nurses’ ability to engage with clients regularly and over time was beneficial in supporting clients to self‐identify certain needs (e.g., substance use or mental illness):

‘‘*that person will say, ‘I'm ready to address my homelessness or my addiction’. Or, ‘can you help me I can't afford to buy cigarettes and want to do some smoking cessation*’’’ (Sharon).

Within this flexible approach, the homeless health nurses occupied spaces that were familiar and accessible to clients: ‘‘*before Covid, [a café] let us work there outside and it just so happened to be over the road from the main pharmacist that gives all the methadone scrips’’* (Hannah). The nurses also ran ‘‘*drop‐in’’* sessions in key community locations to increase their visibility: ‘‘*people can just come and see the nurses [in our support service], get a bit of an MOT [a health check]’’* (Jackie).

In addition, the nurses were well‐positioned to holistically understand clients’ needs and scaffold (i.e., refer clients) to additional services. The nurses played an advocacy role that reduced gaps in healthcare continuity (e.g., maintaining professional involvement following hospital discharge) and assisted clients to access additional support: ‘‘*[the nurses are] bringing new people into the [drug and alcohol] service’’* (Greg). Overall, dedicated nurse‐led homeless healthcare was perceived to be more visible and accessible to clients.

##### Subtheme 1C: Changes in holistic outcomes

The homeless health nurses’ compassion, expertise and trauma‐informed approach was perceived to improve clients’ mental wellbeing, self‐concept and self‐esteem: “*that [both homeless health nurses] accommodated her, met her, and found her to take her to a safe space to do her dressings, made her feel valued*” (Sharon). These changes impacted clients’ motivation towards their physical health, such as improved treatment adherence and proactive help‐seeking:

“*By actually being there every week for her, [the client's] beginning to think ‘do you know what, I think I am worth it. I will put that cream on, or I will take that tablet, I will pick up my methadone script*’.” (Hannah).

Examples of clients attending overdue screening or diagnostic appointments were also provided. Alongside the assumed benefits to physical health from attending healthcare appointments, clients were less likely to experience health‐related worry:

“*If I had gone through the regular GP formal channels, it probably would have taken a lot longer [for the client to receive treatment] … the stress and anxiety that it had caused this individual because of their symptoms, they didn't know whether they had a month to live or whether it was something that wasn't so serious*.” (Katie).

Treating blood‐borne viruses was deemed successful, and the physical health provision was estimated to have reduced hospitalisations: ‘‘*I emailed [the homeless health nurse] … she saw him straight away, gave him some antibiotics, and he's on the mend again. Probably in the past would have meant the hospital’’* (Erin).

Other holistic implications of homeless healthcare were described, including for housing: ‘‘*it's enabled people to sustain their tenancy because it all goes hand in hand with like physical health, mental health, addictions’’* (Jackie). The nurses also supported clients during COVID‐19: ‘‘*[clients] have really struggled with lockdown, and social distancing… the nurses have been able to reinforce [the COVID‐19 guidance]’*’ (Jackie).

#### 3.2.2 Theme 2: The multi‐agency approach of nurse‐led homeless healthcare

This theme describes how professional knowledge shared across the wider multi‐disciplinary team supported clients (subtheme 2A) and the evolution of a multi‐agency approach (subtheme 2B).

##### Subtheme 2A: Client support via professional knowledge‐exchange

Through collaborating with the homeless health nurses ‘‘case by case’ and during multi‐agency meetings, wider partners gained knowledge about mental and physical health and healthcare systems. Professionals then implemented this new‐found knowledge in their own roles: ‘‘*[the nurses] can give us advice on what we should and shouldn't be doing”* (Jackie). The nurses also provided reassurance and guidance to partner organisations whose expertise was not directly in health: ‘‘*I just feel assured when they're there, ‘cause I feel out my depth with mental health*’’ (Colin). Building on this experiential or informal knowledge‐sharing, several professionals from the wider team requested the nurses deliver structured teaching: ‘‘*we would like some more formal training, especially around hep C…basic mental health training would be really helpful*’’ (Jackie).

The addition of nurse‐led‐homeless healthcare had impacted local government professionals, who had increased local intelligence about the needs of people experiencing homelessness, and more understanding of trauma‐informed approaches:

‘‘*they come to our weekly tactical meetings*… *[the nurses] help, I want to say, challenge our thinking, so it makes sure that we are much more insightful as to how people might behave’’* (Donna).

##### Subtheme 2B: Multi‐agency coordination and collaboration

Within nurse‐led homeless healthcare, the collaboration between the mental health and general nurses was positively appraised by various professionals. The nurses conducted joint assessments which enabled clients to talk about mental health: “*dealing with someone's physical health issues, you can lead the conversation into something that's much more sensitive’’* (Donna). Combining each nurse's specialism strengthened holistic support: ‘‘[*the general nurse] could do hep C testing, I can be alongside and then if that person is positive, I can then offer some advice, counselling, support’’* (Fern). Physically working together was also perceived as safer: ‘‘*working alone you're vulnerable’’* (Fern). Alongside the strengths of these partnerships, one possible limitation related to financial cost: ‘‘*there's lots of good reasons why [the general and mental health nurses] need to work together, but…two [senior] nurses going round together is not the most effective use of money*’’ (Barney).

More broadly in the multi‐agency network, the homeless health nurses and other organisations worked in conjunction to support clients to access additional services: *‘‘we are planning together, we're doing work jointly and I think we can be confident that we've had better outcomes for individuals’’* (Donna). This coordination was particularly important during COVID‐19. Several organisations needed to reduce face‐to‐face contact with clients during the pandemic, and so the homeless health nurses (who maintained in‐person delivery) facilitated contact between clients and other professionals working with the client. As an example, the nurses made every effort to avoid clients experiencing gaps in support due to the pandemic: ‘‘*I did the needle exchange training… so that I could take that on the streets… making sure that people weren't reusing needles and syringes*.’’ (Sharon). Despite these challenges of COVID‐19, partnership working was widely appraised as successful and supported by regular online meetings: ‘‘*you're able to get hold of people a bit better [online]’’* (Erin).

#### 3.2.3 Theme 3: Future development of nurse‐led homeless healthcare

Participants proposed various ways to develop nurse‐led homeless healthcare. Findings were related to developing suitable information‐sharing systems and referral pathways (subtheme 3A), recognising the ongoing structural and organisational challenges experienced (subtheme 3B), improving the capacity of homeless healthcare (subtheme 3C) and generating and measuring long‐term impact (subtheme 3D).

##### Subtheme 3A: Developing information‐sharing and referral pathways

Information‐sharing across the multi‐agency team was important to coordinate client support, and data‐sharing protocols were an established first step: ‘‘*we've all got signed consent now from the individuals’’* (Michelle). Information was shared in regular multi‐agency meetings, although remaining up‐to‐date about clients could be challenging: *‘‘you might have a meeting on a Monday and you think, ‘right, I know what's happening with this guy’. And next week it's completely changed*’’ (Barney). Some professionals (e.g., the general homeless health nurses and primary care doctors) could access up‐to‐date shared noting systems, and this improved efficient and accurate communication. However, the mental health and general nurses relied on phone or email updates, so a noting system accessible by all the homeless health nurses was wanted. A major benefit to this would be a reduction in the likelihood of harm to clients, who may otherwise be asked to repeat social and medical histories: *“these are people, generally that aren't used to being brought into an indoor space and being questioned, if you like, unless it's in a very negative way*.” (Sharon).

Partner organisations had largely referred clients to the homeless health nurses informally, via email or telephone: ‘‘*we [voluntary organisation] just ping over an email with as much detail and risk as we've got, a contact number and they'll [the nurses] make contact*’’ (Lisa). However, a referral form had recently been introduced for the mental health nurses. While forms were recognised as important for ‘‘*documentation and monitoring*’’ (Katie), this process restricted responsiveness:

“*You have to make the online referral and then wait for that to be processed [by the organisation] for [the mental health nurse] to make contact and come out. It loses that fluidity*.” (Jackie).

The consensus was therefore to redevelop the referral process to fulfil reporting needs while maintaining a flexible approach.

Finally, relationships between some organisations in the system were perceived to be currently underutilised, which could hinder information‐sharing and referral opportunities. Increased and formalised integration between primary and secondary healthcare and the homeless healthcare provision, particularly at the point of hospital discharge, was also likely to improve care continuity:

‘‘*people go into [intensive care], come round and they self‐discharge and they come out and they're literally back on the street…it's hearsay that I find out, but there should be some pathway*.’’ (Barney).

##### Subtheme 3B: Ongoing organisational and structural challenges

Organisational and structural challenges were identified in relation to the current nurse‐led provision, notably regarding the secondment of the homeless mental health nurses into a voluntary organisation (as illustrated in Figure 1). Differences between the voluntary organisation (and wider voluntary sector) and the statutory sector (i.e., the NHS) were found in the availability of training and supervision, which did not always match professional registration requirements: ‘‘*[the nurses] have a code of professional practice, which the voluntary sector may not be able to follow’’* (Raj). There were also practical issues, such as that the technological resources and systems in the voluntary organisation were not designed to meet the needs of the homeless mental health nurses: ‘‘*the [voluntary organisation] clinical system is not appropriate to capture the work from a mental health point of view’’* (Hannah).

Participants also described that there were conflicting roles and responsibilities between the voluntary and statutory sectors, along with differences in policies and procedures. Taken together, these perceived cultural differences contributed to challenges with the structural arrangements of the nurse‐led mental healthcare provision:

‘‘*the voluntary sector are very much about street level grassroots…we were taking the best of that and mixing it with the expertise from the nurses in the NHS…somehow that doesn't mix so well*’’ (Adrian).

In light of these ongoing structural challenges, participants from various organisations identified that moving the homeless mental health provision into a statutory organisation (i.e. the NHS) may be more appropriate to overcome these challenges: ‘‘*the [general nurses] work very well independently working for [the NHS trust] …that would be better, and [that the nurses] worked with all the other charities the same’’* (Fern). As indicated here, regardless of where nurse‐led homeless healthcare was hosted, it was essential to maintain strong partnerships with the voluntary organisations.

##### Subtheme 3C: Capacity of nurse‐led homeless healthcare

Current capacity was perceived to have been impacted by expansion of the eligibility criteria in year 2 of the pilot (i.e., to all individuals vulnerable housed), which raised particular challenges for offering intensive mental healthcare:

‘‘*there is a challenge with the numbers we've got, with the complexity that we've got, the nurses will not have enough time on their hands. Because it [mental illness] can't be a quick fix*’’ (Donna).

Because of the small number of homeless health nurses, participants perceived that homeless healthcare could be vulnerable to gaps in care continuity if one nurse was unavailable, or if the nurses were unable to cover their assigned geographical area: ‘‘*they are having to cover a very wide area”* (Erin).

The homeless health nurses’ capacity was also impacted by insufficient access to appropriate clinical or therapeutic spaces across some areas of the county, “*I've run round [town] trying to be out of the sight of cameras…I’ve had to do dressing changes in the disabled toilet in [the supermarket]*” (Sharon). Thinking ahead, participants from some partner organisations offered for the homeless health nurses to have better access to organisational spaces and wanted nurses to be ‘‘*working with [our organisation] here as a base’’* (Nic). Another suggestion was the provision of equipped mobile transport, such as an ambulance, that could be utilised across all towns and rural areas to improve person‐centred care:

“*where we could just say to people ‘just pop in the back for a minute and let's have a look at that that wound or that foot’ to give people back their privacy and dignity*.” (Sharon).

##### Subtheme 3D: Generating and measuring long‐term impact

The need to evidence the impact of nurse‐led homeless healthcare was identified. However, an acceptable measure of mental wellbeing was not identified, and quantitative scales used routinely in statutory healthcare were perceived to be less appropriate for use in homeless healthcare: *“the people we work with may not wish to be answering a set of questions every time.”* (Adrian).

To better inform long‐term planning and delivery of nurse‐led homeless healthcare, some participants noted the importance of clear and adequate commissioning. One priority was ‘‘*finding out if it's a permanent service’’* (Barney). In general, many participants widely felt ‘‘*proud that this [homeless healthcare] is happening’’* (Raj) and were eager to support dedicated the homeless healthcare provision to continue and develop: ‘‘*I really hope that they can continue in the months and years to come’’* (Katie).

## 4 DISCUSSION

This evaluation interviewed a range of professionals involved with nurse‐led homeless healthcare in a UK county. The findings reported how the homeless health nurses, specialising in mental or physical healthcare, responded to existing health inequalities, improved health outcomes and offered a novel contribution to a strengthened multi‐agency approach. Challenging areas needing further development largely related to prevailing system and organisational‐level barriers and, if addressed, could lead to enhancements in the delivery of nurse‐led homeless healthcare.

### 4.1 Strengths and outcomes of nurse‐led homeless healthcare

The findings contributed additional evidence for the benefits of nurse‐led homeless healthcare in improving health and reducing health and social inequalities for people who are homeless (Bryar, 2020; Ungpakorn & Rae, 2020). The nurses delivered person‐centred care aligned with a biopsychosocial model likely to support clients’ interacting and diverse unmet needs (Omerov et al., 2020). Positive implications for health extended from aspects of psychological wellbeing, (e.g., improved self‐esteem, confidence, motivation and worry) to physical health outcomes (e.g., symptom alleviation, screening attendance and treatment uptake), including via increased medication adherence and help‐seeking by clients.

Consistent with wider evidence, participants contrasted nurse‐led homeless healthcare with mainstream healthcare, perceived as more inflexible to meet homeless clients’ needs (Gunner et al., 2019; Hauff & Secor‐Turner, 2014; Rae & Rees, 2015). Instead, nurse‐led homeless healthcare appeared to increase accessibility to testing, therapies and treatments owing to full‐time provision that prioritises and advocates for the homeless community (Davies & Wood, 2018; Harney et al., 2019; Su et al., 2015). The nurses’ compassionate approach counteracted clients’ previous negative healthcare experiences, and their compassion was successfully combined with expertise in mental illness and physical health.

Multi‐agency working between the homeless health nurses and voluntary and statutory sectors was successfully aligned with national strategies to integrate health and social care services (NHS England, 2021). Partnership working via case work and multi‐agency meetings supported professionals to exchange knowledge, build client pathways and overcome usual issues with dual diagnosis (Carrà et al., 2015; Gunner et al., 2019). By building on the relational partnerships and collaboration that had developed, the findings also identified ways in which organisational and system‐level factors could be developed to strengthen homeless healthcare.

### 4.2 Moving nurse‐led homeless healthcare forward

Areas requiring further development were associated with contextual and structural factors of homeless healthcare. In particular, a secure shared system between the mental health and general nurses could improve safeguarding of clients and professionals, avoid unnecessary repeated history‐taking, and promote care continuity (Mathioudakis et al., 2016). Participants also advocated for more formalised pathways between homeless healthcare and primary and secondary care. Wider evidence supports that people who are homeless are often discharged from hospital into settings that do not promote recovery or access to continued care and so, where locally available, homeless healthcare must be effectively integrated into healthcare transitions (Canham et al., 2019). Secondary healthcare providers have a legal duty to refer rough sleepers to local governments, and so integrating dedicated homeless healthcare into this pathway is likely to be a feasible way of improving client care (Ministry of Housing, Communities & Local Government, 2018).

The mental health nurses’ secondments into the voluntary organisation were beneficial in implementing the flexible, street‐based approach already adopted by the voluntary sector. However, conflicting practical and cultural approaches between the sectors were also reported, consistent with wider evidence about such differences in organisational norms, practice, knowledge and outcome measurements (Renedo, 2014). In addition, participants raised the importance of improving capacity in the nurses’ roles, particularly in the context of a larger and more rural county. Because of the complexities associated with homelessness, appropriately low caseloads (resulting from appropriate referral criteria) are likely to be important in ensuring care continuity and intensive provision (Ponka et al., 2020). Assertive community treatment approaches are characterised by multi‐disciplinary working and low caseloads offering community‐based, intensive support (Coldwell & Bender, 2007). Evidence has supported this approach for homeless individuals because of improved housing stability, mental health and psychiatric outcomes (Moledina et al., 2021). Advances in nurse‐led homeless healthcare must therefore adopt structures that support professionals with diverse roles and responsibilities, and facilitate intensive and multi‐agency support to clients (Parsell et al., 2020).

Finally, participants indicated that inequalities in healthcare access could prevail while adequate clinical and therapeutic spaces were lacking. Participants advocated for mobile transport or increased utilisation of community spaces to enhance client privacy and confidentiality (Kiser & Hulton, 2018). Similarly, understanding of the longer‐term commissioning plans would assist stakeholders to plan and prepare adequately in relation to local homeless healthcare. As well as forming a standard part of implementation and evaluation efforts, it is crucially important to optimise the role that nurses play to support and sustain nurse‐led healthcare for the homeless community. A summarised list of recommendations is provided in File S1.

### 4.3 Potential limitations

Due to challenges acquiring confidential spaces for client interviews during COVID‐19, this evaluation did not directly incorporate the voices of clients. While professionals agreed about how nurse‐led homeless healthcare had positively affected clients, it is not known how far professional perceptions align with client experiences, and this work is ongoing by the research team. Though beyond the scope of this study, examination of the impact of homeless healthcare on outcomes such as hospitalisation and rough sleeping would contribute beneficial evidence.

Participants from a range of organisations contributed constructive thoughts to this evaluation. However, it was recognised that the interview context may have influenced participant's contributions, and some stakeholders (such as police and probation services) were not successfully recruited. The findings also do not intend to be applied to other homeless health services, particularly where local systems and infrastructure may differ.

Considerations should also be granted to the context of COVID‐19. The homeless community, including professionals, were impacted by closures of community spaces, routine disruption and changed formats of support services (Kaur et al., 2021; Parkes et al., 2021). These contexts shaped the roles of the nurses during this time, as they increased accommodation visits, facilitated support between clients and other services, and attended online multi‐agency meetings. Therefore, while nurse‐led homeless healthcare was operational prior to the pandemic, there were multi‐faceted implications of COVID‐19.

## 5 CONCLUSION

This qualitative evaluation reinforced the benefits of dedicated homeless healthcare in reducing health inequalities through accessible, flexible and person‐centred care. Multi‐agency partnership working was essential to holistically support clients and professionals. Important organisation and system‐level recommendations based on the findings included developing robust organisational pathways, structural considerations related to scope and capacity, and increasing availability of clinical spaces, with these recommendations expected to further enhance access to healthcare and health promotion for individuals experiencing homelessness.

## CONFLICT OF INTEREST

The authors do not declare any conflict of interest for this work.

## AUTHOR CONTRIBUTION

EF, DL and LB contributed to the study design. Interviews were conducted by LB. Analysis was conducted by LB and MW. All authors interpreted the findings and contributed to manuscript drafts and revisions.

## Supporting information

Supplementary Material

## Data Availability

Data are not publicly available to preserve participant anonymity.

## References

[hsc13778-bib-0001] Aldridge, R. W. , Story, A. , Hwang, S. W. , Nordentoft, M. , Luchenski, S. A. , Hartwell, G. , Tweed, E. J. , Lewer, D. , Vittal Katikireddi, S. , & Hayward, A. C. (2018). Morbidity and mortality in homeless individuals, prisoners, sex workers, and individuals with substance use disorders in high‐income countries: A systematic review and meta‐analysis. The Lancet, 391(10117), 241–250. 10.1016/S0140-6736(17)31869-X PMC5803132 29137869

[hsc13778-bib-0002] Braun, V. , & Clarke, V. (2006). Using thematic analysis in psychology. Qualitative Research in Psychology, 3(2), 77–101. 10.1191/1478088706qp063oa

[hsc13778-bib-0003] Braun, V. , & Clarke, V. (2020). One size fits all? What counts as quality practice in (reflexive) thematic analysis? Qualitative Research in Psychology, 1–25, 10.1080/14780887.2020.1769238

[hsc13778-bib-0004] Braun, V. , & Clarke, V. (2021). To saturate or not to saturate? Questioning data saturation as a useful concept for thematic analysis and sample‐size rationales. Qualitative Research in Sport, Exercise and Health, 13(2), 201–216. 10.1080/2159676X.2019.1704846

[hsc13778-bib-0005] Bryar, E. R. (2020). Homeless Health Innovation Funding Programme: Evaluation Report. The Queen’s Nursing Institute, https://www.qni.org.uk/wp‐content/uploads/2020/08/HH‐Innovation‐Funding‐Programme‐Evaluation‐2020.pdf

[hsc13778-bib-0006] Canham, S. L. , Davidson, S. , Custodio, K. , Mauboules, C. , Good, C. , Wister, A. V. , & Bosma, H. (2019). Health supports needed for homeless persons transitioning from hospitals. Health & Social Care in the Community, 27(3), 531–545. 30011102 10.1111/hsc.12599

[hsc13778-bib-0007] Carrà, G. , Bartoli, F. , Brambilla, G. , Crocamo, C. , & Clerici, M. (2015). Comorbid addiction and major mental illness in Europe: A narrative review. Substance Abuse, 36(1), 75–81. 10.1080/08897077.2014.960551 25222286

[hsc13778-bib-0008] Coldwell, C. M. , & Bender, W. S. (2007). The effectiveness of assertive community treatment for homeless populations with severe mental illness: A meta‐analysis. American Journal of Psychiatry, 164(3), 393–399. 17329462 10.1176/ajp.2007.164.3.393

[hsc13778-bib-0009] Crane, M. , Cetrano, G. , Joly, L. , Coward, S. , Daly, B. , Ford, C. , Gage, H. , Manthorpe, J. , & Williams, P. (2018). Mapping of specialist primary health care services in England for people who are homeless: Summary of findings and considerations for health service commissioners and providers. King’s College London. https://www.kcl.ac.uk/scwru/res/hrp/hrp‐studies/HEARTH/HEARTH‐study‐Mapping‐SummaryReport‐2018.pdf

[hsc13778-bib-0010] Cream, J. , Fenney, D. , Williams, E. , Baylis, A. , Dahir, S. , & Wyatt, H. (2020). Delivering health and care for people who sleep rough: Going above and beyond. The King’s Fund. https://www.kingsfund.org.uk/sites/default/files/2020‐02/Delivering‐health‐care‐people‐sleep‐rough.pdf

[hsc13778-bib-0011] Davies, A. , & Wood, L. J. (2018). Homeless health care: Meeting the challenges of providing primary care. Medical Journal of Australia, 209(5), 230–234. 10.5694/mja17.01264 30157413

[hsc13778-bib-0012] Elwell‐Sutton, T. , Fok, J. , Albanese, F. , Mathie, H. , & Holland, R. (2017). Factors associated with access to care and healthcare utilization in the homeless population of England. Journal of Public Health, 39(1), 26–33. 10.1093/pubmed/fdw008 26896508

[hsc13778-bib-0013] Fazel, S. , Geddes, J. R. , & Kushel, M. (2014). The health of homeless people in high‐income countries: Descriptive epidemiology, health consequences, and clinical and policy recommendations. The Lancet, 384(9953), 1529–1540. 10.1016/S0140-6736(14)61132-6 PMC4520328 25390578

[hsc13778-bib-0014] Field, H. , Hudson, B. , Hewett, N. , & Khan, Z. (2019). Secondary care usage and characteristics of hospital inpatients referred to a UK homeless health team: A retrospective service evaluation. BMC Health Services Research, 19(1), 10.1186/s12913-019-4620-1 PMC6868755 31752857

[hsc13778-bib-0015] Flanagan, S. M. , & Hancock, B. (2010). ’Reaching the hard to reach’—Lessons learned from the VCS (voluntary and community Sector). A qualitative study. BMC Health Services Research, 10(1), 92. 10.1186/1472-6963-10-92 20377850 PMC2856561

[hsc13778-bib-0016] Fletcher, A. J. (2017). Applying critical realism in qualitative research: Methodology meets method. International Journal of Social Research Methodology, 20(2), 181–194. 10.1080/13645579.2016.1144401

[hsc13778-bib-0017] Gunner, E. , Chandan, S. K. , Marwick, S. , Saunders, K. , Burwood, S. , Yahyouche, A. , & Paudyal, V. (2019). Provision and accessibility of primary healthcare services for people who are homeless: A qualitative study of patient perspectives in the UK. British Journal of General Practice, 69(685), e526. 10.3399/bjgp19X704633 PMC6650120 31307999

[hsc13778-bib-0018] Harney, B. L. , Whitton, B. , Lim, C. , Paige, E. , McDonald, B. , Nolan, S. , Pemberton, D. , Hellard, M. E. , & Doyle, J. S. (2019). Quantitative evaluation of an integrated nurse model of care providing hepatitis C treatment to people attending homeless services in Melbourne, Australia. International Journal of Drug Policy, 72, 195–198. 10.1016/j.drugpo.2019.02.012 30981613

[hsc13778-bib-0019] Hauff, A. J. , & Secor‐Turner, M. (2014). Homeless health needs: Shelter and health service provider perspective. Journal of Community Health Nursing, 31(2), 103–117. 24788048 10.1080/07370016.2014.901072

[hsc13778-bib-0020] Kaur, S. , Jagpal, P. , & Paudyal, V. (2021). Provision of services to persons experiencing homelessness during the COVID‐19 pandemic: A qualitative study on the perspectives of homelessness service providers. Health & Social Care in the Community. 10.1111/hsc.13609 PMC8653035 34668258

[hsc13778-bib-0045] Kiser, T. , & Hulton, L. (2018). Addressing health care needs in the homeless population: a new approach using participatory action research. SAGE Open, 8(3), 2158244018789750.

[hsc13778-bib-0021] Lucas, S. , Archard, P. J. , Tangen, J. , & Murphy, D. (2018). Arrangements for adult service users who are homeless in English mental health trusts. Mental Health Review Journal, 23(1), 64–71. 10.1108/MHRJ-03-2017-0017

[hsc13778-bib-0022] Luchenski, S. , Maguire, N. , Aldridge, R. W. , Hayward, A. , Story, A. , Perri, P. , Withers, J. , Clint, S. , Fitzpatrick, S. , & Hewett, N. (2018). What works in inclusion health: Overview of effective interventions for marginalised and excluded populations. The Lancet, 391(10117), 266–280. 10.1016/S0140-6736(17)31959-1 29137868

[hsc13778-bib-0023] Magwood, O. , Hanemaayer, A. , Saad, A. , Salvalaggio, G. , Bloch, G. , Moledina, A. , Pinto, N. , Ziha, L. , Geurguis, M. , Aliferis, A. , Kpade, V. , Arya, N. , Aubry, T. , & Pottie, K. (2020). Determinants of implementation of a clinical practice guideline for homeless health. International Journal of Environmental Research and Public Health, 17(21), 7938. 33138054 10.3390/ijerph17217938 PMC7663114

[hsc13778-bib-0024] Mariano, M. A. , & Harmon, M. J. (2019). Living libraries: Nurse integration in interprofessional homeless health care team. Public Health Nursing, 36(2), 172–177. 10.1111/phn.12561 30467899 PMC7379664

[hsc13778-bib-0025] Mathioudakis, A. , Rousalova, I. , Gagnat, A. A. , Saad, N. , & Hardavella, G. (2016). How to keep good clinical records. Breathe (Sheffield, England), 12(4), 369–373. 28210323 10.1183/20734735.018016 PMC5297955

[hsc13778-bib-0044] Ministry of Housing, Communities & Local Government (2018). A guide to the duty to refer. https://www.gov.uk/government/publications/homelessness‐duty‐to‐refer/a‐guide‐to‐the‐duty‐to‐refer

[hsc13778-bib-0026] Mitchell, M. S. , León, C. L. K. , Byrne, T. H. , Lin, W.‐C. , & Bharel, M. (2017). Cost of health care utilization among homeless frequent emergency department users. Psychological Services, 14(2), 193–202. 28481604 10.1037/ser0000113

[hsc13778-bib-0027] Moledina, A. , Magwood, O. , Agbata, E. , Hung, J.‐H. , Saad, A. , Thavorn, K. , & Pottie, K. (2021). A comprehensive review of prioritised interventions to improve the health and wellbeing of persons with lived experience of homelessness. Campbell Systematic Reviews, 17(2), e1154. 10.1002/cl2.1154 PMC8356292 37131928

[hsc13778-bib-0028] National Health Service England (2021). What are integrated care systems? https://www.england.nhs.uk/integratedcare/what‐is‐integrated‐care/

[hsc13778-bib-0029] Omerov, P. , Craftman, Å. G. , Mattsson, E. , & Klarare, A. (2020). Homeless persons’ experiences of health‐ and social care: A systematic integrative review. Health & Social Care in the Community, 28(1), 1–11. 10.1111/hsc.12857 31524327

[hsc13778-bib-0030] Parkes, T. , Carver, H. , Masterton, W. , Falzon, D. , Dumbrell, J. , Grant, S. , & Wilson, I. (2021). ‘They already operated like it was a crisis, because it always has been a crisis’: A qualitative exploration of the response of one homeless service in Scotland to the COVID‐19 pandemic. Harm Reduction Journal, 18(1). 10.1186/s12954-021-00472-w PMC7927775 33658042

[hsc13778-bib-0031] Parsell, C. , Clarke, A. , & Vorsina, M. (2020). Evidence for an integrated healthcare and psychosocial multidisciplinary model to address rough sleeping. Health & Social Care in the Community, 28(1), 34–41. 31452354 10.1111/hsc.12835

[hsc13778-bib-0032] Ponka, D. , Agbata, E. , Kendall, C. , Stergiopoulos, V. , Mendonca, O. , Magwood, O. , Saad, A. , Larson, B. , Sun, A. H. , Arya, N. , Hannigan, T. , Thavorn, K. , Andermann, A. , Tugwell, P. , & Pottie, K. (2020). The effectiveness of case management interventions for the homeless, vulnerably housed and persons with lived experience: A systematic review. PLoS One, 15(4), e0230896. 32271769 10.1371/journal.pone.0230896 PMC7313544

[hsc13778-bib-0033] Public Health England (2019). Homelessness: Applying all our health. https://www.gov.uk/government/publications/homelessness‐applying‐all‐our‐health/homelessness‐applying‐all‐our‐health

[hsc13778-bib-0034] Public Health England (2021). Public Health Profiles. https://fingertips.phe.org.uk/search/homeless#page/0/gid/1/pat/6/par/E12000005/ati/102/iid/11501/age/‐1/sex/‐1/cat/‐1/ctp/‐1/yrr/1/cid/4/tbm/1

[hsc13778-bib-0035] Queen, A. B. , Lowrie, R. , Richardson, J. , & Williamson, A. E. (2017). Multimorbidity, disadvantage, and patient engagement within a specialist homeless health service in the UK: An in‐depth study of general practice data. BJGP Open, 1(3), bjgpopen17X100941. 10.3399/bjgpopen17X100941 PMC6262212 30564673

[hsc13778-bib-0036] Rae, B. E. , & Rees, S. (2015). The perceptions of homeless people regarding their healthcare needs and experiences of receiving health care. Journal of Advanced Nursing, 71(9), 2096–2107. 10.1111/jan.12675 25916241

[hsc13778-bib-0037] Renedo, A. (2014). Care versus control: The identity dilemmas of UK homelessness professionals working in a contract culture. Journal of Community & Applied Social Psychology, 24(3), 220–233.

[hsc13778-bib-0038] Snelling, C. (2017). Right to home: Rethinking homelessness in rural communities. Institute for Public Policy Research. https://www.ippr.org/files/2017‐06/1498563647_right‐to‐home‐a4‐report‐170627.pdf

[hsc13778-bib-0039] Stefanowicz, M. , Feldman, B. , & Robinson, J. (2021). House calls without walls: street medicine delivers primary care to unsheltered persons experiencing homelessness. The Annals of Family Medicine, 19(1), 84. 33431400 10.1370/afm.2639 PMC7800740

[hsc13778-bib-0040] Su, Z. , Khoshnood, K. , & Forster, S. H. (2015). Assessing impact of community health nurses on improving primary care use by homeless/marginally housed persons. Journal of Community Health Nursing, 32(3), 161–169. 26212468 10.1080/07370016.2015.1057082

[hsc13778-bib-0041] Tong, A. , Sainsbury, P. , & Craig, J. (2007). Consolidated criteria for reporting qualitative research (COREQ): A 32‐item checklist for interviews and focus groups. International Journal for Quality in Health Care, 19(6), 349–357. 10.1093/intqhc/mzm042 17872937

[hsc13778-bib-0042] Ungpakorn, R. , & Rae, B. (2020). Health‐related street outreach: Exploring the perceptions of homeless people with experience of sleeping rough. Journal of Advanced Nursing, 76(1), 253–263. 31588583 10.1111/jan.14225

[hsc13778-bib-0043] Warwickshire County Council (2021). Preventing Homelessness in Warwickshire: A multi‐agency approach. https://api.warwickshire.gov.uk/documents/WCCC‐1980322935‐1894

