# Supplementary material for: Nurse‐led mental and physical healthcare for the homeless community: A qualitative evaluation
Source: Health Soc Care Community. 2022 Mar 9;30(6):2282–91. doi: 10.1111/hsc.13778 (PMC10078647; doi:10.1111/hsc.13778)
Supplement: Supplementary file 1 — Supplementary Material [file HSC-30-2282-s001.docx]

**Supplementary File 1**

## Recommendations based on findings

- Continue to optimise nurse-led homeless healthcare provision which is flexible, accessible, visible and delivers person-centred care using outreach and community approaches.
- Sustain the multi-agency approach and continue regular multi-disciplinary team meetings (and consider if sustain remote/online format beyond COVID-19 restriction easing to optimise attendance).
- Formalise pathways and increase awareness of the provision to services and health professionals (e.g. GPs) across the county.
- Continue to work closely with partner organisations, including in a street outreach capacity, to holistically support clients with a range of needs.
- Develop referral pathways that enable responsive and timely care for clients with direct and relational communications between the nurses and referring agencies.
- Ensure data sharing protocols, processes, and client consent procedures are established for new partner organisations collaborations.
- Consider the feasibility of shared IT systems or noting procedures for the homeless health nurses.
- Provision of scheduled training sessions by the nurses to educate and raise awareness amongst partner organisations about homeless client health.
- Define eligibility criteria for the homeless health service which is appropriate for the number of full-time nurses, and geographical area, so that sufficiently intensive support can be offered.
- Develop the provision of clinical or therapeutic spaces across the county which are appropriate and safe for clients and nurses (using existing or new resources such as a dedicated mobile unit).
- Ensure that employment structures for nurses facilitate professional registration, including clinical supervision and continuing professional development.
- Develop means or measures to record client outcomes in a consistent and appropriate manner to monitor change.
- Examine longer-term commissioning to support service planning and development.
